# Investigating the reinforcing mechanism and optimized dosage of pristine graphene for enhancing mechanical strengths of cementitious composites†

**DOI:** 10.1039/d0ra07639b

**Published:** 2020-11-25

**Authors:** Van Dac Ho, Ching-Tai Ng, Togay Ozbakkaloglu, Ramesh U. Karunagaran, Farzaneh Farivar, Andy Goodwin, Craig Mc Guckin, Van Duong Ho, Dusan Losic

**Affiliations:** School of Civil, Environmental and Mining Engineering, The University of Adelaide Australia alex.ng@adelaide.edu.au; School of Chemical Engineering, The University of Adelaide Australia dusan.losic@adelaide.edu.au; ARC Research Hub for Graphene Enabled Industry Transformation, The University of Adelaide Australia; Ingram School of Engineering, Texas State University USA; First Graphene Ltd Suite 3, 9 Hampden Road Nedlands WA 6009 Australia; University of Architecture Ho Chi Minh City Vietnam

## Abstract

The proposed reinforcing mechanism and optimized dosage of pristine graphene (PRG) for enhancing mechanical, physicochemical and microstructural properties of cementitious mortar composites are presented. Five concentrations of PRG and two particle sizes are explored in this study. The results confirmed that the strength of the mortars depends on the dosage of PRG. The PRG sizes have a significant influence on the enhancement rate of mechanical strengths of the mortars, whereas they do not have a significant influence on the optimized PRG dosage for mechanical strengths. The PRG dosage of 0.07% is identified as the optimized content of PRG for enhancing mechanical strengths. The reinforcing mechanism of PRG for cement-based composites is mostly attributed to adhesion friction forces between PRG sheets and cementitious gels, which highly depends on the surface area of PRG sheets. The larger surface area of PRG sheets has a larger friction area associated with cementitious gels suggested to be one of favorable parameters for enhancing mechanical strengths with graphene additives.

## Abstract 2

The proposed reinforcing mechanism and optimized dosage of pristine graphene (PRG) for enhancing mechanical, physicochemical and microstructural properties of cementitious mortar composites are presented.

## Graphical abstract

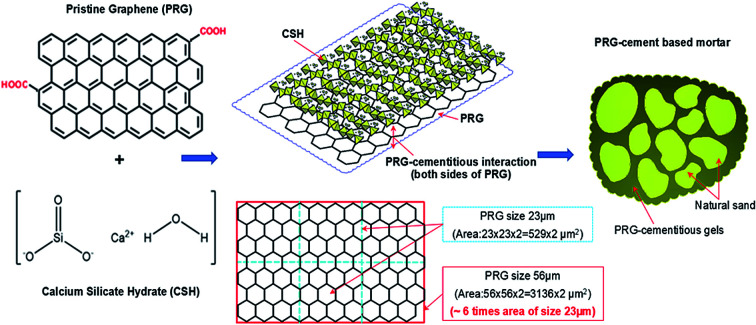

## 1. Introduction

Cementitious composites are the most common construction materials because of their low cost, availability, and high strength in compression. Nevertheless, cementitious composites are weak in tensile strength, and poor in resisting crack propagation and corrosive environments, *e.g.* sulphate ions and chloride ions.^1,2^ To improve these drawbacks, studies showed benefits of using reinforcement such as steel, carbon or plastic fibres^3,4^ to impede the propagation of microcracks, or additives with nanomaterials such as SiO_2_ and TiO_2_,^5,6^ carbon nanofibers and carbon nanotubes^7–9^ to accelerate the cement hydration process and create materials with denser microstructures.^10–13^ However, these supplementary materials are zero or one-dimensional materials with limited performance in bonding and arresting cracks at the nanoscale, and unable to efficiently enhance the reinforcement.^7–9,14^

Recently studies have shown that two-dimensional materials such as graphene derivatives have a good potential for enhancing performances of cement composites because of their high properties and aspect ratios.^15,16^ The applications of different graphene derivatives with different properties (*e.g.* graphene oxide (GO), reduced graphene oxide (rGO), and pristine graphene (PRG)) in cementitious composites have been explored in the literature.^17–19^ GO was the most attractive graphene material due to its favourable functional groups on the surface (*e.g.* carboxyl and hydroxyl), which provides higher reactivity with cement and high dispersion in water. Many studies reported that the addition of GO into cement composites could significantly improve their mechanical properties.^17–19^ Kang *et al.*^20^ reported that incorporating GO into cement-based mortars improved 28 day compressive and flexural strength by approximately 32% (at 0.05% GO) and 20% (at 0.1% GO). Zhao *et al.*^21^ showed that incorporating 0.022% GO into cement mortars produced a 34.1% and 30.4% enhancement in 28 day compressive and flexural strength, respectively. The influence of the dosage and size of GO on the microstructure of cementitious mortar composites was also reported in the literature.^22,23^ Lv *et al.*^23^ showed that as the size of GO decreased from 430 nm to 72 nm, the enhancement rates of 28 day compressive and flexural strengths could be increased from 29.5% and 30.7% to 38.2% and 51.9%, respectively.

The mechanism of the enhancement of GO-cement based composites is accounted for the considerable effect of oxygen-functional groups of graphene oxide on the cement matrix. This shows that smaller sizes of GO will have more oxygen-functional groups than larger sizes of GO, which leads to stronger adhesion forces between the functional groups and cementitious gels. Based on GO research, several studies reported the reinforcing mechanism of mechanical strengths of GO-cement based composites which were mainly governed by chemical reactions between hydroxyl and carboxyl groups of GO and the mediating Ca^2+^ ions from calcium silicate hydrate of cementitious gels. This results in a space network structure in the cement matrix that supports the load transfer efficiency in cementitious composites.^14,24^ Compared with GO, PRG is a remarkably different graphene material with a very limited level of oxygen groups, higher crystallinity, lower defects, and significantly stronger mechanical properties.^25,26^ Therefore, it has attracted significant research interests in using the PRG in cementitious composites.^17,18,27–30^ The limitation in water dispersion of PRG sheets (PRGs) has been addressed in recent studies by using superplasticizer and ultrasonication methods.^27,30–32^ It has been shown that a small amount of PRG has great potential to enhance the strength of PRG-cement composites.^27–30,32,33^ Wang *et al.*^30^ reported that 0.05% of PRG could enhance 7 & 28 day flexural compressive strengths of cement-based mortars by 23.5% & 16.8% and 7.5% & 1.3%, respectively. Besides, the influence of the dosage of pristine graphene on the strength of cementitious mortar composites has been reported in recent studies.^27,28,33^ Baomin and Shuang^33^ investigated the use of four different dosages of PRG in cement paste and reported that the optimal PRG dosage of 0.06% could increase the 28 day compressive and flexural strength of the cement paste approximately 11% and 27.8%, respectively. Tao *et al.*^28^ studied a combination of cement-based mortars and five dosages of PRG. The results showed that the mortar with PRG of 0.05% could enhance 28 day compressive strength about 8.3% and 28 day flexural strength about 15.6%. However, when the dosages of PRG over 0.05%, the strengths started reducing because of the agglomerations of PRGs. In our previous work, we performed a comprehensive investigation of the dosage dependence of PRG-cement based mortars using seven dosages of pristine graphene.^27^ The results revealed that compressive and tensile strengths at 7 days & 28 days of the mortar containing 0.07% pristine graphene significantly improved by 36.8% & 34.3% and 25.3% & 26.9%, respectively.

These studies only showed the dependence of the strength of cementitious composites on pristine graphene dosages. The reinforcing mechanism of pristine graphene on the strength of cementitious composites has not been well understood. In addition to the dosage of PRG, there are several parameters of PRGs, such as particle sizes, level of defects and numbers of layers, can affect the performance of PRG-cement based composites. As reported in ref. 34–36, these parameters have a considerable effect on mechanical properties of polymer composites. However, there were very limited studies on the effects of these parameters on mechanical strengths of PRG and cementitious composites. To date, only few studies have applied molecular dynamics simulation methods to investigate the interaction between PRGs and cementitious gels at the atomic level.^37,38^ The outcomes of these studies showed that the pull-out behaviour of PRG in cementitious gels was governed by interfacial interaction and crack surface adhesion forces of PRG–cementitious gels. Although these studies provided the initial knowledge of the incorporation of pristine graphene into cementitious gels, there is still a lack of experimental confirmation on the reinforcing mechanism of PRGs in cementitious composites.

On the other hand, studies on GO-cement showed a wide range of optimum dosages of GO (*i.e.* from 0.01% to 1%) for improving the strengths of the composites,^17–19^ which is one of the bottlenecks in practical applications. The dosage dependence of mechanical properties of cementitious composites prepared with pristine graphene on the dosages of PRG showed a much better convergence in the optimal PRG dosage range (*i.e.* from 0.05% to 0.07%) even though they differed from mix designs and PRG materials used.^27,28,33^ These studies showed great potential for applying a small amount of PRG additives in construction materials to improve their mechanical strengths and other properties. However, very limited studies have been done to explore the consistency of these optimum PRG dosages used in cement composites which can support future studies on designing tests for investigating other properties of cement-based materials by graphene additives. Moreover, pristine graphene materials have better crystalline structures and properties than graphene oxide and they are now produced with high quality and low costs. As a result, it is expected that industrially produced PRG materials will be more acceptable additives for improving the properties of construction materials.

This study aims at providing an in-depth investigation of the aforementioned issues with a focus on revealing the reinforcing mechanism and optimized dosage of industrially manufactured PRG materials for enhancing the strengths of cement-based mortars. The impact of the dosage of pristine graphene with two different particle sizes on mechanical and microstructural properties of cementitious mortar composites are explored, compared, and presented in this research. From the findings and comprehensive analyses of this study, we provide new inputs toward a better knowledge in the proposed reinforcing mechanism and optimized dosage of pristine graphene on the strength of cementitious mortars. This paper not only provides a better knowledge of incorporating PRG into cementitious composites, but they also show the great potential for low-cost industrially produced PRG materials for addressing current drawbacks of cementitious materials.

## 2. Experimental section

### 2.1. Materials

PRG materials manufactured by First Graphene Ltd in Perth, Australia, were used in this study (Table 1). These PRG materials were produced by an electrochemical process, which is a unique manufacturing process using electrochemistry to exfoliate PRGs with a few layers, large particle sizes and low defects from graphite that are not achievable by other methods (*e.g.* thermal or chemical methods from rGO). The general schematic mechanism of PRG materials produced by an electrochemical exfoliation process is illustrated in Fig. S1 (ESI†). The binder was ordinary Portland cement (OPC) with general purpose and its chemical composition (Table 2) abided by the Australian Standard AS 3972-2010.^39^ Natural sand was used as the fine-aggregate for all mortar mixes, which had maximum particle sizes of 2.36 mm. Table 3 presents the properties of the superplasticizer used in this study that was MasterGlenium SKY 8100 – a polycarboxylic ether polymer in order to improve the dispersion of PRG in aqueous solution, which was abided by the Australian Standard AS 1478.1-2000.^40^

**Table 1:** Physical properties of PRGs used in this study

ID	Average particle	Thickness	Purity	Poured bulk density
	size (μm)	(nm)	(%)	(g cm^−3^)
S1	56 ± 12	1–3	98.3	∼0.12
S2	23 ± 10	1–3	98.3	∼0.11

**Table 2:** Chemical properties of Portland cement

Compounds	OPC (%)
CaO	63.28
SiO_2_	19.95
Al_2_O_3_	4.79
Fe_2_O_3_	3.14
MgO	2.03
Na_2_O	0.29
K_2_O	0.4
SO_3_	2.69
P_2_O_5_	0.04

**Table 3:** Properties of superplasticizer

pH	Boiling temperature (°C)	Density at 20 °C (kg dm^−3^)	Flash point (°C)	Vapour pressure at 20 °C (hPa)	Solid content (mass, %)
6.4	≥ 100	1.06	> 100	23	30.7

### 2.2. Preparation of the mortar composites

The design mixes are given in Table 4. As shown in the table, a total of 9 unique mortar mixes were performed, including five different concentrations and two different sizes of PRG, *i.e.* 0%, 0.05%, 0.07%, 0.1% and 0.3% mixes and average PRG diameters of 56 μm (S1) and 23 μm (S2). Table 4 shows the labels used for the mixes. S1 and S2 refer to PRG with an average size of 56 μm and 23 μm, respectively. The number after that indicates the PRG dosage in each mix, which is calculated by the weight of the binder. For example, S1-0.05 indicates the PRG-cement based mortar prepared with a PRG size 56 μm and a PRG content of 0.05%.

**Table 4:** The design mixes of the mortars

Mix	PRG^a^ (%)	PRG size (μm)	Water/cement ratio	Cement (kg m^−3^)	Water (kg m^−3^)	PRG (kg m^−3^)	Sand (kg m^−3^)	Superplasticizer (kg m^−3^)
Plain	—	—	0.485	527	255.6	0.0	1448	1.4
S1-0.05	0.05	56	0.485	527	255.6	0.3	1448	1.4
S1-0.07	0.07	56	0.485	527	255.6	0.4	1448	1.4
S1-0.1	0.1	56	0.485	527	255.6	0.5	1448	1.4
S1-0.3	0.3	56	0.485	527	255.6	1.6	1448	1.4
S2-0.05	0.05	23	0.485	527	255.6	0.3	1448	1.4
S2-0.07	0.07	23	0.485	527	255.6	0.4	1448	1.4
S2-0.1	0.1	23	0.485	527	255.6	0.5	1448	1.4
S2-0.3	0.3	23	0.485	527	255.6	1.6	1448	1.4

a The percentage of PRG based on cement weight.

The procedures described below were applied to prepare PRG-cement based mortars. The aqueous solution was first prepared, and it consisted of water, superplasticizer and pristine graphene. Sonication was then carried out using Ultrasonication UIP1000hdT for 30 minutes. After that, the sonicated aqueous solution was gradually added into dry mixings, which included OPC and natural sand mixed within four minutes, for five minutes. A vibration table was used to vibrate these specimens for one minute to remove the entrapped air, then covered by wet fabrics to contain the moisture loss, and demolded after 24 hours of curing at room temperature. After that, they were cured in a fog room with a temperature of 23 ± 2 °C until the testing ages.

### 2.3. Test methods

Mechanical strengths (compression and tension) of cementitious composites were determined at 7 and 28 days according to ASTM standards C109/C109M-07 (ref. 41) and C307-03,^42^ respectively. These tests were performed to investigate the influence of the dosage of pristine graphene with two different particle sizes on mechanical and microstructural properties of cementitious mortar composites. The mechanical strengths of each design mix were determined by calculating the average values of three samples. The variance method was performed to assess the statistically significant difference in mechanical strengths of the mortars containing PRG in the optimum dosage range.

Scanning electron microscopy (SEM) and energy-dispersive X-ray spectroscopy (EDX) were obtained by using FEI Quanta 450 to analyze surface morphologies and elemental compositions of materials. The particle size distribution was performed by using the Mastersizer 2000-Malvern to analyze the particle size of PRGs used in this study. The Rigaku MiniFlex 600 X-ray diffractometer was used for X-ray diffraction (XRD) analyses to find the mineralogical characteristics of hydration products of cementitious composites and the distances between layers in pristine graphene sheets. The XRD was carried out at conditions 40 kV and 15 mA, 2*θ* = 5°–80° at 0.02° step size. Thermogravimetric analysis (TGA) of PRG samples was conducted with Mettler Toledo TGA/DSC 2 (the heating rate at 10 °C min^−1^ under air atmosphere with a flow rate of 60 ml min^−1^). Raman spectroscopy (LabRAM HR Evolution, Horiba Jvon Yvon Technology, Japan) with a 532 nm laser (mpc3000) as the excitation source in the range of 500–3500 cm^−1^ was utilized to study the vibrational characteristics of carbon materials. All spectra were collected at an integration time of 10 s for 3 accumulations using a 100× objective lens with a spot size of 100 μm. Raman map was performed for a 20 μm × 20 μm area with 2 μm steps and Raman spectrum of overall 121 points were collected for each sample. Nicolet 6700 was used for Fourier transform infrared spectroscopy (FTIR) analyses to identify functional groups of materials.

## 3. Results and discussion

### 3.1. Characteristics of pristine graphene sheets

**Fig. 1:**
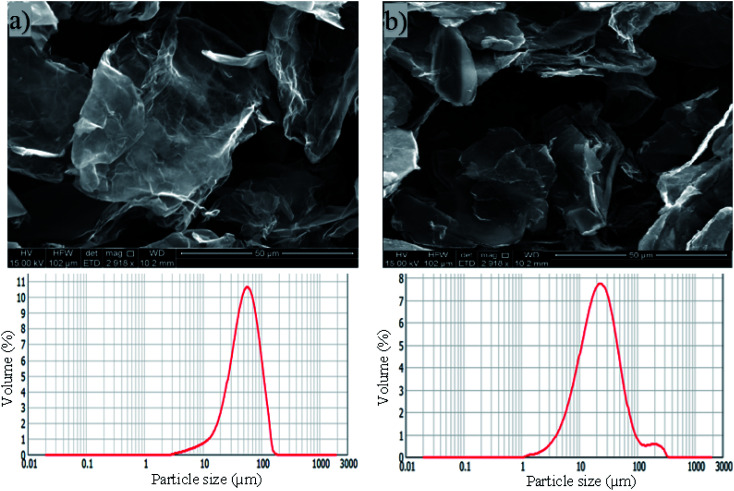
SEM images and particle size distribution of PRG: (a) size 56 μm (S1), (b) size 23 μm (S2).

The physical properties and morphology of industrially manufactured pristine graphene with two different graphene sheets particle sizes used in the study are characterized and summarized in Table 1 and Fig. 1, respectively. The irregular shapes of PRGs were observed in SEM images, as shown in Fig. 1. These SEM images and particle size distributions shown in Fig. 1(a) and (b) present a considerable difference in the particle size of 56 μm (S1) and 23 μm (S2), respectively. Table 1 also shows that the physical properties of these two PRGs are similar and only different in particle sizes.

**Fig. 2:**
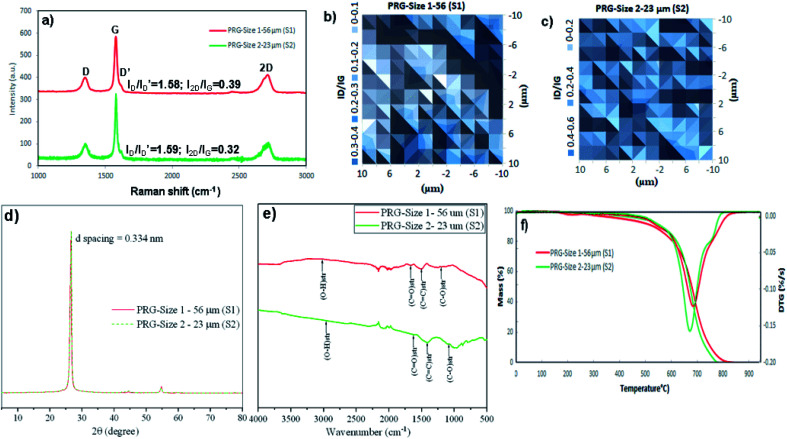
Characterization of two PRG samples: (a) Raman spectra, (b) and (c) *I*_D_/*I*_G_ peak ratio mappings, (d) XRD patterns, (e) FTIR spectra, (f) TGA/DTG graphs.

Fig. 2(a)–(c) presents the Raman spectra and Raman *I*_D_/*I*_G_ map of both PRG samples. The Raman peak at the 2D band can be used to indicate the number of layers in the graphene samples based on the frequency shift and the shape of the 2D peak. It can be seen in Fig. 2(a) that a narrow and symmetric 2D band at 2709 cm^−1^ is observed in these PRG samples which are different from graphite materials that have a broad and asymmetric 2D peak located at 2719 cm^−1^. Besides, their relative intensity ratios *I*_D_/*I*_D′_ and *I*_2D_/*I*_G_ shown in Fig. 2(a) are respectively 1.58 & 1.59 and 0.39 & 0.32, which are below 3.5 and 1. Moreover, the distribution histogram plots of relative intensity ratios *I*_D_/*I*_G_ of both PRG sizes obtained from the mapping study are mostly below 0.4–0.6 (Fig. 2(b) and (c)). These combined results confirm that both PRG samples are high-quality products with low defects, and they mostly consist of few-layer sheets of graphene,^43–46^ which are critical for their optimized performance in cement-based composites.

Fig. 2(d) presents XRD graphs showing typical peaks for both PRG samples at position 2*θ* = 26.64°. Based on the Bragg's law, the *d*-spacing between layers of both PRGs was 0.334 nm, the same as the properties of graphite materials.^47,48^ This shows that the high crystalline structure and quality of pristine graphene materials in this investigation. FTIR spectra in Fig. 2(e) show the major characteristic bands for both PRG sizes at: 1000 cm^−1^ to 1240 cm^−1^ is attributed to C–O groups; 1700 cm^−1^, and from 2500 cm^−1^ to 3300 cm^−1^ are referred to C=O stretching and O–H stretching. These functional groups present the existence of carboxylic acids (*i.e.* COOH) in both PRGs, which are likely in limited numbers at the edge of PRG structure. The stretching vibration from 1300 cm^−1^ to 1600 cm^−1^ corresponds to C=C groups. These are consistent with FTIR results on pristine graphene materials in the literature, which shows minor oxygen groups at edges of their structures.^49,50^Fig. 2(f) shows a typical TGA–DTG graph of PRG samples. The figure shows the maximum thermal decomposition peak of both PRG samples is about 700 °C which is different from GO, rGO,^49,51,52^ presenting the high quality of PRG materials used in this study. The typical decomposition patterns of TGA–DTG curves of GO and rGO are also provided in Fig. S2 (ESI†) for the comparison purpose.

It is important to note that, in this study, two industrially produced PRG samples with the same manufacturing process were used, which are high-quality products, have similar physicochemical properties, and their main difference is particle sizes. Therefore, the influence of other parameters of PRG materials (*e.g.* levels of defects, and numbers of layers) on the strength of cementitious mortar composites is negligible, and the main influence parameter of PRG materials on the different mechanical results of the mortars containing two PRG samples in this study accounts for the difference in PRG sizes.

### 3.2. Mechanical properties of mortar mixes

**Fig. 3:**
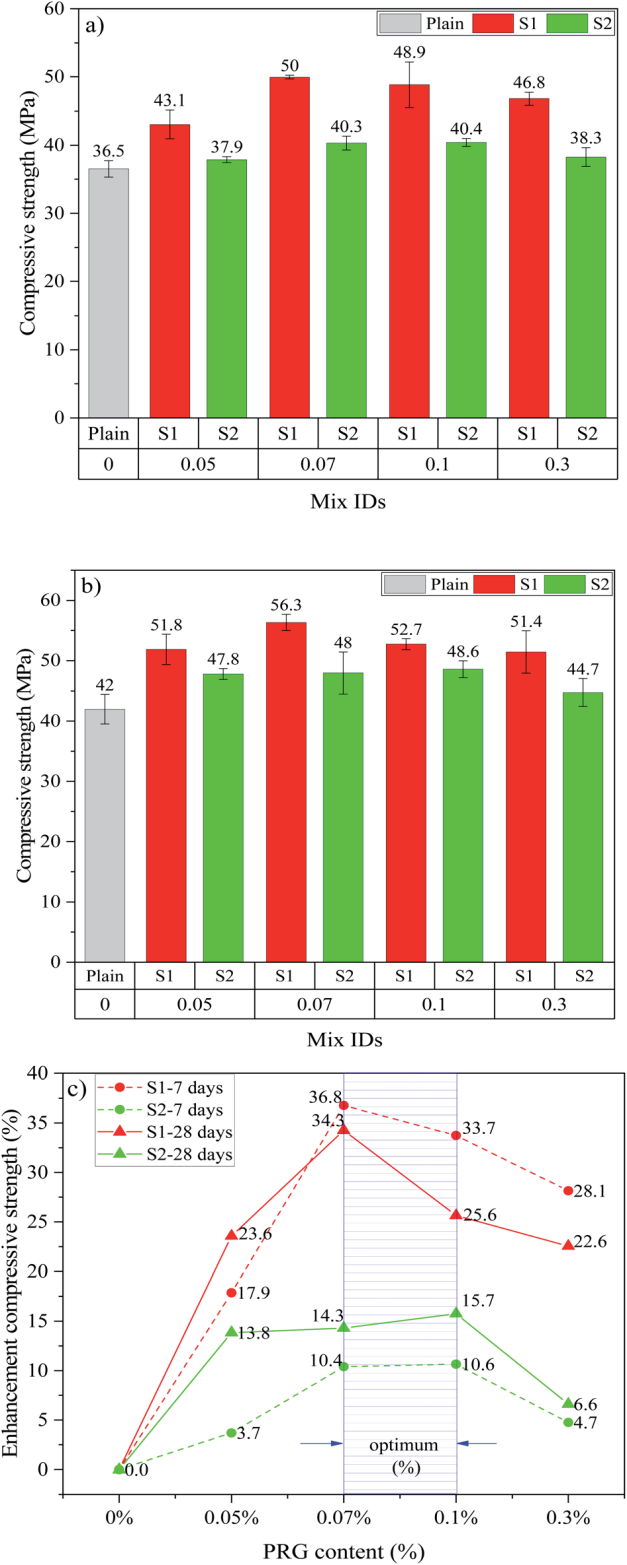
Compressive strength at: (a) 7 days, (b) 28 days; (c) enhancement compressive strength at 7 and 28 days of different PRG-cement based mortars.

Fig. 3 shows the compressive strengths and strength enhancements of cement-based mortars with different PRG dosages and sizes at 7 and 28 days. As shown in Fig. 3(a)–(c), the addition of PRG to cementitious composites increases the 7 day and 28 day compressive strengths of the mortars. The mixes containing larger PRG size S1 and smaller PRG size S2 have the optimal PRG dosage at 0.07% and 0.1%, respectively. When PRG is used beyond the optimal dosage, the compressive strengths of the mortars in both PRG sizes start decreasing. As shown in the figure, at the optimal dosage of 0.07% PRG, the 7 day and 28 day compressive strengths of the mix containing larger PRG size S1 (*i.e.* S1-0.07) are approximately 50 MPa and 56.3 MPa, respectively. These are 36.8% and 34.3% higher than the corresponding strengths of the plain mortar (36.5 MPa and 42 MPa, respectively). For the mortar containing smaller PRG size S2 at the optimal dosage of 0.1%, the 7 day and 28 day compressive strengths are 40.4 MPa and 48.6 MPa, respectively, which represent only a 10.6% and 15.7% respective increase compared to the plain mortar.

**Fig. 4:**
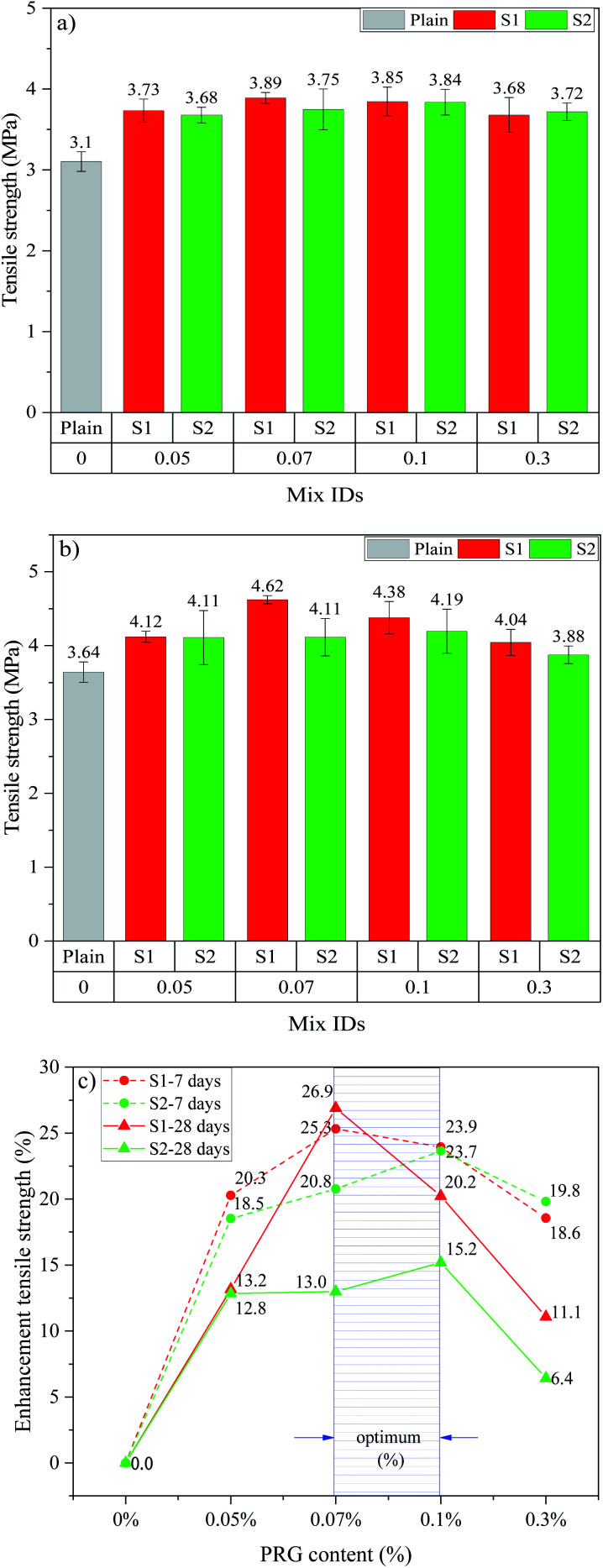
Tensile strength at: (a) 7 days, (b) 28 days; (c) enhancement tensile strength at 7 and 28 days of different PRG-cement based mortars.

The tensile strengths and strength enhancements of cementitious mortar composites with the different dosage and size of pristine graphene at 7 and 28 days are shown in Fig. 4. From Fig. 4(a)–(c), it can be seen that incorporating PRG into cementitious composites increases the 7 day and 28 day tensile strengths of the mortars. The trend in tensile strengths is similar to that in compressive strengths for both PRG samples at both ages. As shown in the figure, at the optimal dosage of 0.07% PRG, the 7 day and 28 day tensile strengths of the mix containing larger PRG size S1 are 3.89 MPa and 4.62 MPa, respectively, which respectively improve approximately 25.3% and 26.9% compared with the plain mortar at these testing days (3.10 MPa and 3.64 MPa). However, for the mix containing smaller PRG size S2 at the optimal dosage of 0.1% PRG, the 7 day and 28 day tensile strengths are 3.84 MPa and 4.19 MPa, respectively, which represent a 23.7% and 15.2% respective increase compared to the plain mortar.

The reduction in enhancement rates of mechanical strengths of the mortars when using PRG beyond the optimal dosage can stem from the poor dispersion of PRG suspension due to the van der Waals forces between PRGs. This leads to the agglomeration of PRGs and the formation of multi-layers PRGs, resulting in the hindrance to the enhancement of PRGs to the hydration process, as well as their interaction with cementitious gels.^27,49,53^

Figs. 3(c) and 4(c) show the optimal PRG range for both compression and tension at 7 day and 28 day ages of PRG-cement based mortars. Both pristine graphene samples are in a relatively low dosage range of from 0.07% to 0.1%, and the strength improvement rates are between 10.4% and 36.8%. The variance test was performed to assess if the difference in the strength of mortars containing PRG in the optimal range (*i.e.* 0.07% and 0.1%) to be statistically significant. To do this, the variance analysis based on the theory of the null hypothesis with the significant level of 0.05 was tested (the details of this method can be seen in ref. 54 and 55). The results of the analysis of variance test are shown in Table 5. It is evident from the table that the difference between S1-0.07 and S1-0.1 at 28 day compressive strength is statistically significant (*i.e. P*-value = 0.018 < 0.05). However, there are no statistically significant differences in tensile and compressive strengths at curing ages of 7 and 28 days of the other mixes between 0.07% and 0.1% (*i.e. P*-values >0.05). Moreover, for the PRG dosage of 0.07%, the enhancement rates of 7 day & 28 day compressive strengths and tensile strengths of the mix containing larger PRG size S1 are approximately 3.5 & 2.4 times and 1.2 & 2.1 times more than those of the mix containing smaller PRG size S2, respectively. From those analytic results, it can be concluded that the PRG sizes have a significant effect on the enhancement rates of mechanical strengths of the mortars, whereas they do not have a significant influence on the optimized PRG dosage for mechanical strengths of the mortars. Therefore, the pristine graphene dosage of 0.07% is identified as the optimized content of PRG for enhancing the strength of cementitious mortar composites for both sizes. The reinforcing mechanism of pristine graphene on the strengths of the mortars will be discussed in detail in Sections 3.3 and 3.4.

**Table 5:** Analysis of variance tests for evaluating the difference in 7 day and 28 day mechanical strengths of the mortars containing PRG in the optimal dosage range (0.07% PRG and 0.1% PRG)

Difference of levels	Difference of means	*T*-Value	Adjusted *P*-value	Evaluate significant differences
S1-0.07–S1-0.1 (7 day compression)	1.110	0.57	0.596	No
S1-0.07–S1-0.1 (7 day tension)	0.043	0.39	0.716	No
S1-0.07–S1-0.1 (28 day compression)	3.615	3.85	0.018	Yes
S1-0.07–S1-0.1 (28 day tension)	0.243	1.86	0.136	No
S2-0.1–S2-0.07 (7 day compression)	0.092	0.14	0.897	No
S2-0.1–S2-0.07 (7 day tension)	0.090	0.52	0.630	No
S2-0.1–S2-0.07 (28 day compression)	0.610	0.28	0.793	No
S2-0.1–S2-0.07 (7 day tension)	0.080	0.35	0.742	No

### 3.3. Physicochemical and microstructural characterizations of mortar mixes

Complementary XRD, FTIR, and SEM-EDX characterizations were performed to examine the influence of the different dosage and size of pristine graphene on the physicochemical and microstructure characteristics of the composites. The three different PRG concentrations were selected for analysis. They are 0%, 0.07% and 0.3%, which represent the plain mix, the mix with the optimized, and highest considered PRG dosage, respectively. However, XRD and FTIR analysis of smaller PRG size S2 were only presented at 0.07% PRG content for the comparison purpose.

#### 3.3.1. XRD and FTIR characterizations

There are four main components of the OPC binder, *i.e.* tricalcium silicate or alite (C_3_S), dicalcium silicate (C_2_S), tricalcium aluminate (C_3_A), tetracalcium ferroaluminate (C_4_AF), and a small amount of gypsum. The hydration products of the cement matrix resulting from chemical reactions between these components and water^56,57^ can be described by the following equation:

1 (C_3_S, C_2_S, C_3_A, C_4_AF) + gypsum + H_2_O → calcium silicate hydrate (CSH) + portlandite (CH) + sulphoaluminates (most ettringite (Aft)) + part of monosulphoaluminate

**Fig. 5:**
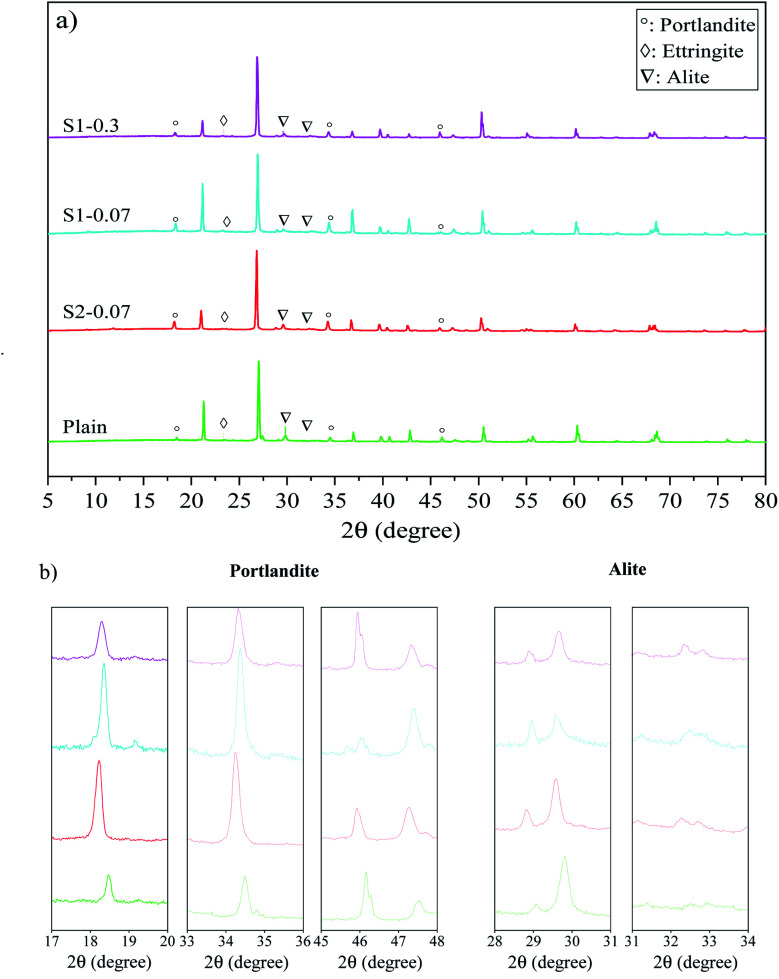
XRD patterns of: (a) different PRG-cement based mortars at 28 days, (b) portlandite and alite detailed from (a) (PRG-cement mortar with size S2 only presented at 0.07% PRG concentration for the comparison purpose).

Fig. 5 presents XRD patterns of different mortar mixes (*i.e.* the plain, S1-0.07, S2-0.07, S1-0.3) at 28 days of curing age. As shown in eqn (1), the production of the Portland cement hydration process consists of CSH gels, CH and Aft. Among them, CSH gels are the main part contributing to mechanical strengths of cementitious composites. Therefore, samples with a larger amount of CSH gels can have better strength properties in composites. In XRD analysis, although there are some difficulties in identifying CSH phases that are often as amorphous phases,^22,58,59^ the content of CSH gels and the hydration degree of the binder can be estimated by the content of portlandite and un-hydrated cement particles (*e.g.* C_3_S, C_2_S).^58,59^ The XRD spectra of all samples were standardized at the peak of 26.7° to ensure the amount of natural sand in specimens equal.^22,59,60^

It can be seen in Fig. 5(a) that although the samples containing pristine graphene show similar spectra with the plain mortar, they have different intensities, which might cause differences in their mechanical properties. From Fig. 5(a) and (b), the peaks of portlandite phases can be identified at 18.2°, 34.2° and 47.1°;^59,61^ and the intensities of these peaks are different from each mix. The highest value is observed at S1-0.07, followed by S1-0.3, S2-0.07, and the plain. This reveals that the hydration degree of cement paste of the mixes containing PRG is higher than that of the plain mix, which is consistent with previous research on PRG-cement composites.^30,59^ In addition, the figures also show that the scattering angles at 29.5° and 32.3° of un-hydrated alite^58,61^ of these mixes have different intensities, which is the highest in the plain mix and followed by the PRG-cement samples. This can be attributed to the beneficial effect of PRG on the cement hydration process, which might lead to the creation of more CSH gels in the cement matrix.^22,58,59^ This is in agreement with the observed trends of the mechanical results of the mixes analyzed in Section 3.2.

**Fig. 6:**
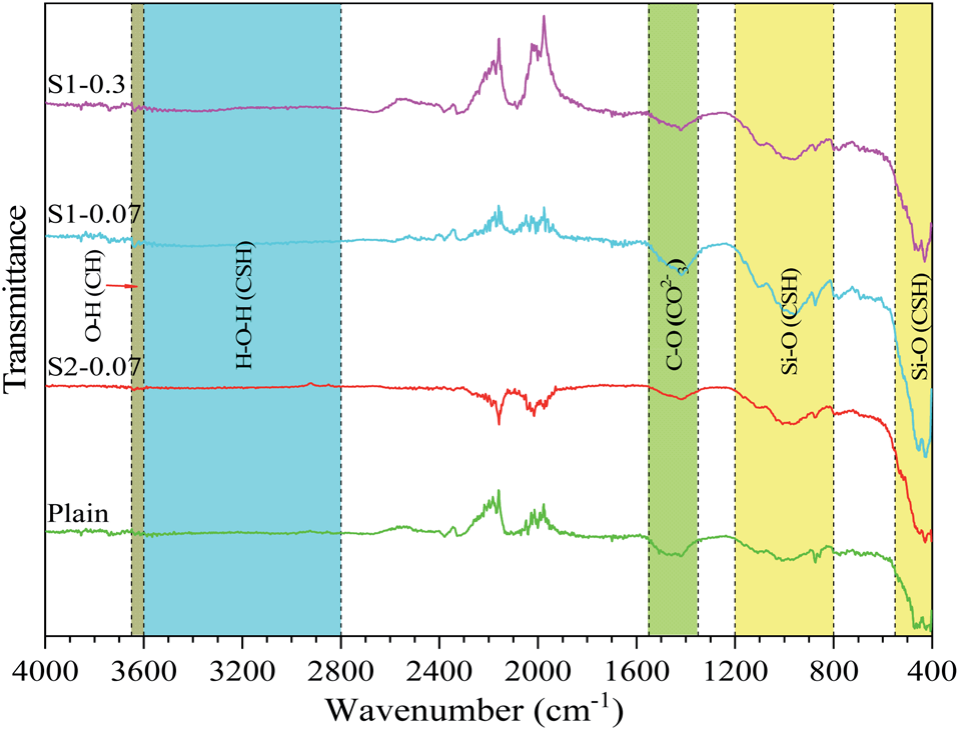
FTIR of different PRG-cement based mortars at 28 days (PRG-cement mortar with size S2 only presented at 0.07% PRG concentration for the comparison purpose).

The FTIR spectra of the different mixes (*i.e.* the plain, S1-0.07, S2-0.07, S1-0.3) at the 28 day testing are shown in Fig. 6. The figure shows that the spectrum of these samples is similar. This means no new specific groups observed when adding PRG. The group bands ranged 400–550 cm^−1^ and 800–1200 cm^−1^ are attributed to Si–O bonds in the CSH gels.^62,63^ The band ranged from 2800 to 3600 cm^−1^ represents O–H groups in H_2_O belonging to CSH gels.^62–64^ The narrow band in the range of about 3600–3650 cm^−1^ is attributed to portlandite, *i.e.* O–H bonds.^62,65^ The band of 1350–1550 cm^−1^ is attributed to C–O bonds in calcium carbonate.^63,64^Fig. 6 also shows that although these mixes have similar spectra, the spectral intensities representing CSH gels and portlandite in these mixes are different. This indicates that the mortars with pristine graphene materials have stronger intensities than the plain mix and the strongest intensity can be observed in the S1-0.07 mix. This may be due to the higher cement hydration degree in the mixes containing pristine graphene, leading to the enhancement in mechanical strengths of those mixes compared with the plain. This is in agreement with the results of mechanical strengths, and XRD shown above.

#### 3.3.2. SEM characterizations

**Fig. 7:**
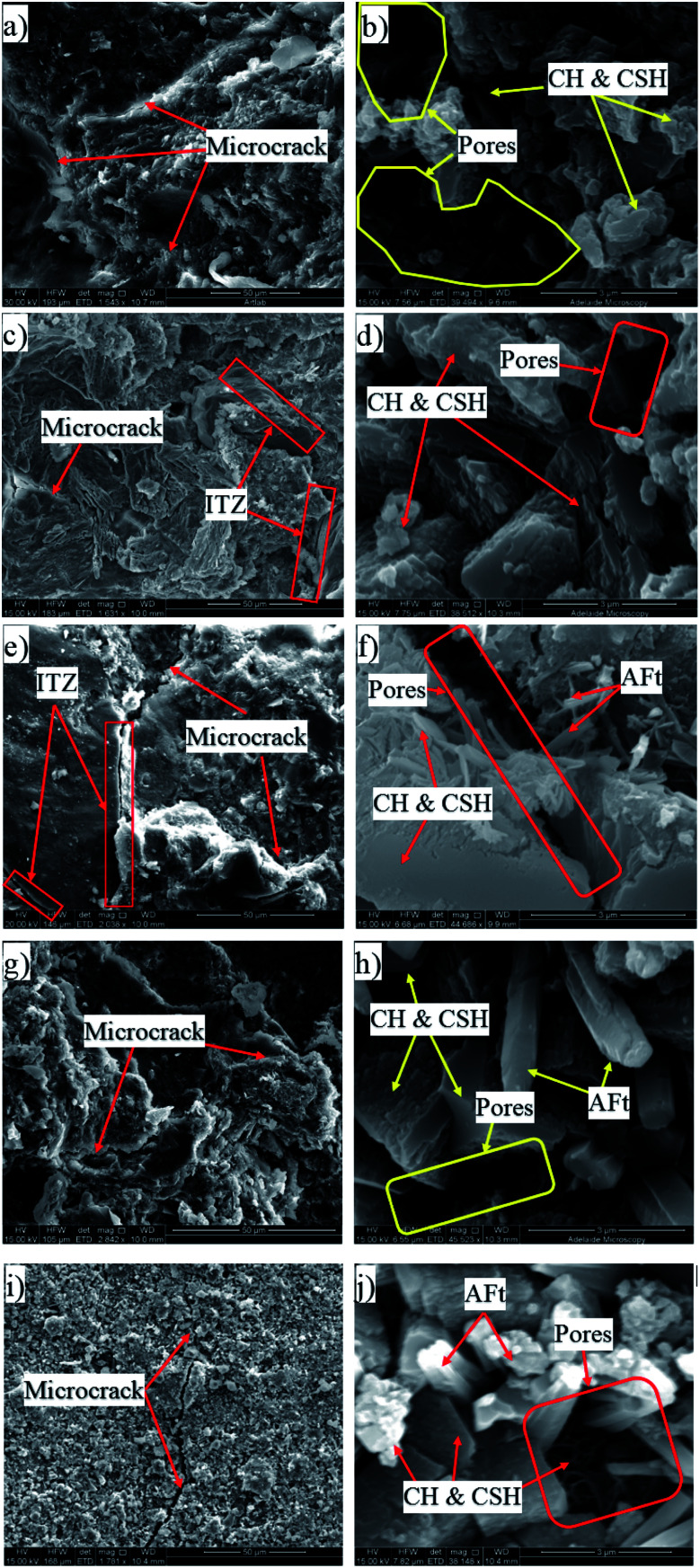
SEM images of different PRG-cement based mortars at 28 days: (a and b) plain (control), (c and d) S1-0.07, (e and f) S2-0.07, (g and h) S1-0.3 and (i and j) S2-0.3 (for each mix, 50 μm and 3 μm magnification corresponds to the former and latter figure respectively).

Fig. 7(a)–(j) shows a series of SEM images of observed microcrack patterns and crystals in different PRG-cement based mortars at the 28 day testing, *i.e.* the plain mortar, S1-0.07, S2-0.07, S1-0.3, and S2-0.3. As shown in the figure, although these mixes have similar components in their structures (*e.g.* CSH, CH, Aft and pores), the distribution and compaction of these components at the microscale are different. The plain mix (Fig. 7(a) and (b)) exhibits higher content of pores and density of microcracks in its microstructure compared to the other mixes. This explains the reason for the lower strengths of the plain mix than those of the mixes prepared with pristine graphene. It can be seen in Fig. 7(c)–(j) that, for a given PRG size, the mixes prepared with the larger PRG size (S1) exhibit better microstructure patterns than those with the smaller PRG size (S2). The crystal content and compactness of the PRG-cement samples are also altered by different PRG dosages for both PRG sizes, which shows the densest microstructure at 0.07% PRG content and followed by 0.3%. As shown in Fig. 7(c)–(f) (*i.e.* at the optimized dosage of 0.07% PRG), the SEM images of the mix containing the larger PRG size (Fig. 7(c) and (d)) are not only more compact in microstructure but it also has denser interfacial transition zones (ITZ) between cementitious gels and fine aggregates than that of the mix containing the smaller PRG size (Fig. 7(e) and (f)). This can contribute to more efficient stress distribution and better inhibition of crack propagation in the structure of the S1 series, resulting in improvements in their mechanical properties,^14,22,30^ and this will be discussed further in Section 3.4.

### 3.4. The reinforcing mechanism of PRG for enhancing the strength of cementitious composites

**Fig. 8:**
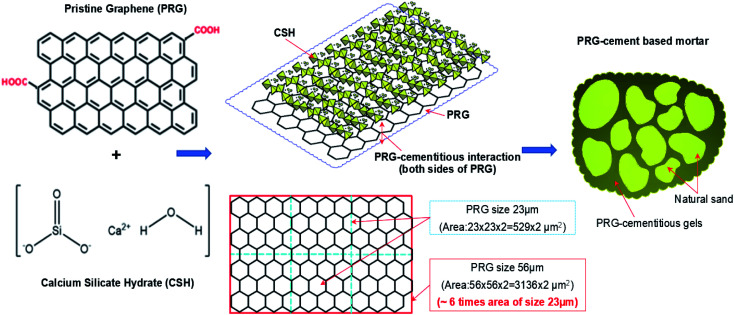
The outline of the proposed mechanism for the formation and enhancement of cementitious gels by PRGs.

The strengths of traditional cementitious composites depend on the strengths of Portland cementitious gels, which are formed by the chemical reaction between cement powder and water. The most important product of the cement hydration process is CSH gels, which contribute most of the strength of Portland cementitious gels.^2,66^ Similar to traditional cement mortar, the strengths of PRG and cementitious mortar composites are governed by PRG–cementitious gels, which are created by the interaction between PRG structure and Portland cementitious gels (CSH gels). Fig. 8 outlines a general illustration of the proposed mechanism showing the interaction of PRG and CSH structures as a key parameter for the enhancement of PRG–cementitious gels in PRG-cement based mortars. As mentioned in the Introduction section, for GO-cement based composites, the reinforcing mechanism of their mechanical properties was proposed as a result of chemical reactions between the oxygen-functional groups (*i.e.* hydroxyl and carboxyl groups) of GO and the mediating Ca^2+^ ions from cementitious gels, resulting in the formation of strong interference bonds between GO and cementitious gels. This improves the space network structure in the cement matrix that supports the load transfer efficiency in structures, resulting in the improvement of mechanical properties of cement composites^14,24^ However, the level of these oxygen-functional groups at the edge of the PRG structure is very limited, and hence, their contribution to strength enhancement of PRG–cementitious gels is less significant. Moreover, as discussed in Section 3.1, both PRG samples have the same manufacturing process, high-quality products and similar physicochemical properties, and are only different in particle sizes. Consequently, the main factor to reinforce the strength of PRG–cementitious gels must be related to the interaction between basal planes of PRGs and CSH gels, which depend on surface areas of PRGs. This means PRGs with larger particle sizes will have larger basal plane areas to interact with CSH gels around. This leads to a stronger connection between them in cement matrix. This finding is significantly supported by the considerable difference in mechanical results of PRG-cement based mortars between the larger PRG size 56 μm (S1) and smaller PRG size 23 μm (S2) as discussed in Section 3.2.

The increase in mechanical strengths of PRG-cement mortars can be explained (which is also supported by the findings presented in next paragraphs): (1) part from the improvement of cementitious gels due to the closer distance between the particles of the cement binder caused by van der Waals forces between PRGs;^17^ (2) the most important part to reinforce PRG–cementitious gels is proposed to be contributed by the adhesion friction forces between surface areas of PRGs and CSH gels: these adhesion friction forces are a combination of crack surface adhesion forces (which was created by atoms near crack surfaces during the pull-out process^38^), and friction forces between surface areas of PRGs and CSH gels, which depend on particle sizes of PRGs that will increase with an increase in graphene size. This was also demonstrated in the study conducted by Chen *et al.*^38^ using Molecular Dynamic (MD) simulation to investigate the interaction mechanism of PRGs (with low surface roughness properties) and CSH gels. The benefit of PRG sizes to enhance PRG–cementitious gels is clearly supported by the experimental results of this study. At the optimized dosage of 0.07%, the strength of 28 day compression and tension of the larger PRG size S1 mix are 2.4 and 2.1 times higher than those of the smaller PRG size S2 mix, respectively. This is because the larger PRG size has larger contact surface areas, which contribute to a better adhesion friction force compared with the smaller PRG size. It is also noted that both PRG samples have similar thicknesses and densities (Table 1), so there is no significant difference in their specific surface areas (*i.e.* unit with m^2^ g^−1^) at the same dosage. However, they are significantly different from the contact surface area of each of PRGs with CSH gels (*i.e.* the area of the large PRG size 56 μm (S1) is approximately 6 times as equal as that of smaller the PRG size 23 μm (S2), as shown in Fig. 8).

**Fig. 9:**
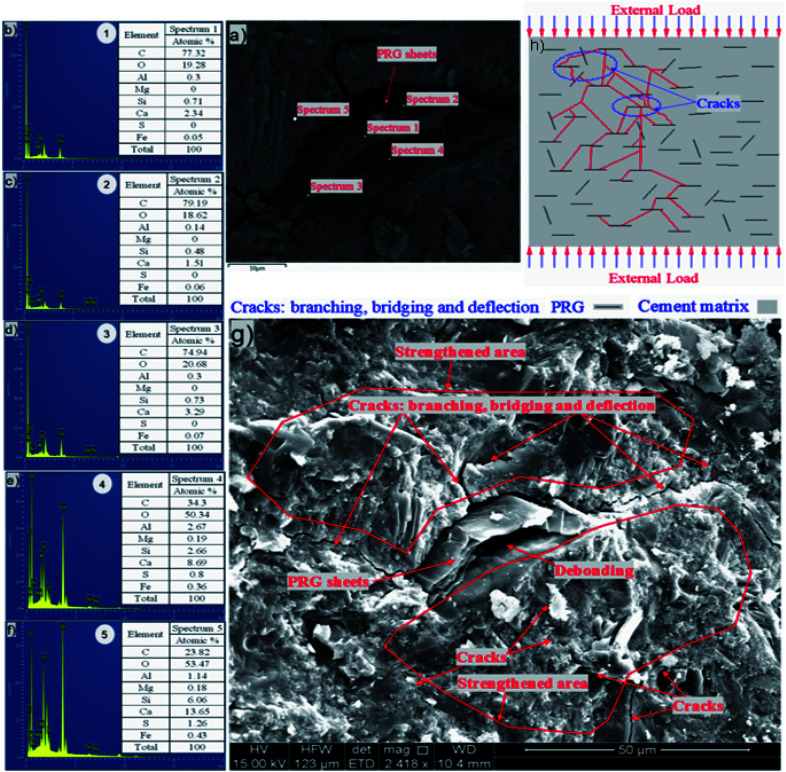
(a–f) Energy dispersive X-ray results confirm the combination of PRGs and cement gels in the cement matrix; (g) the detailed outlines of the supporting of PRGs to enhance properties of cementitious gels when sustaining external loads; (h) the outline of crack paths of PRG-cement based composites under external loads.

**Fig. 10:**
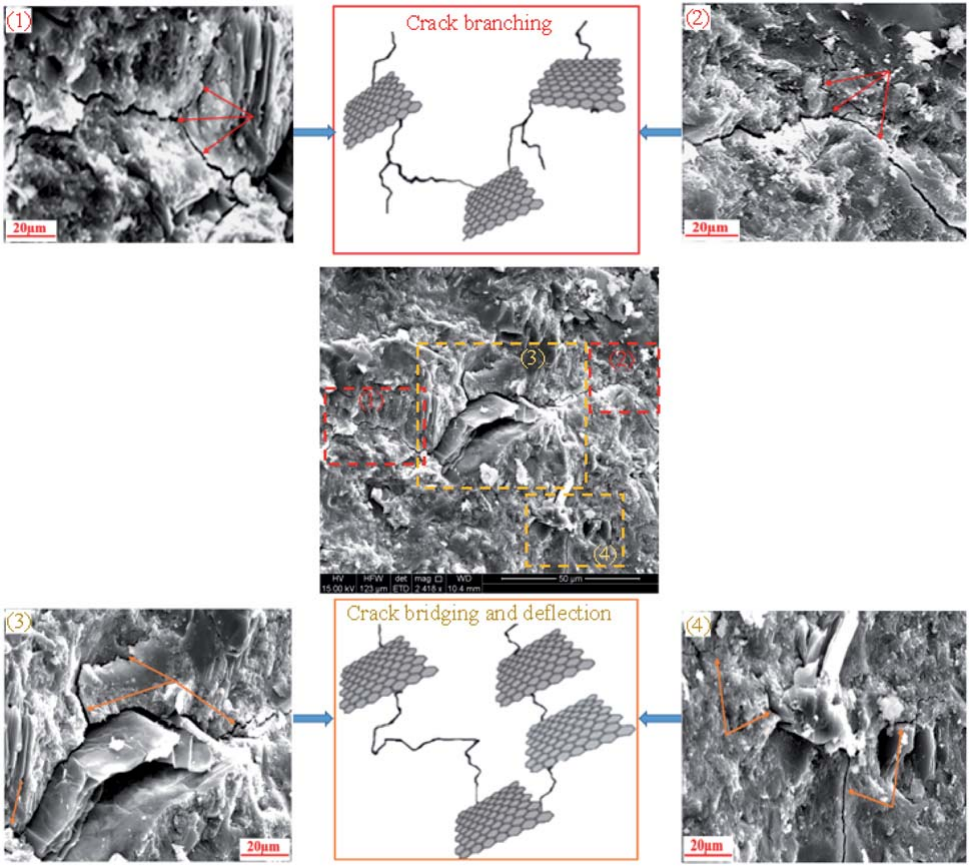
Some details with illustrations to explain the propagation of cracks in the cement matrix.

The investigation of the interface of PRGs and cementitious gels and the propagation of microcracks in the cement matrix was performed and the results are presented in Fig. 9 and 10. As can be seen in Fig. 9(a)–(f), the EDX results indicate that the carbon contents of spectrums 1, 2, and 3 are dominant and much higher than the other spectrums nearby (*i.e.* spectrums 4 and 5). This confirms the cement matrix containing a combination of PRGs and cementitious gels. Fig. 9(g) depicts the detailed outline of the reinforcing mechanism and crack propagation in the cement matrix due to PRG additives. The figure also shows that the combination of PRGs and CSH gels can enhance cementitious gels around PRGs and create interlocked PRG–cementitious gels in a space network structure, resulting in the effectiveness of stress distribution. Figs. 9(g) and 10 also present that PRGs can increase the path of crack developments through crack bridging, crack branching, and crack deflection. This contributes to the benefit of the reduction of crack widths in structures. As a result, it can be said that PRGs with the larger size can create larger interaction areas with CSH gels, and hence, leading to larger strengthened areas. This is more beneficial for their interlocks in the cement matrix compared to the smaller size. This finding is consistent with the important role of PRG sizes on adhesion friction forces of PRG–cementitious gels as discussed before in the previous paragraph.

Moreover, as discussed in Section 3.2, the enhancement strength rates of the mortars start decreasing when PRG is used over the optimum dosage due to the agglomeration of PRGs in the cement matrix, which can also be explained from the SEM images in Fig. 9(g). From the figure, it can be figured out that when the agglomeration of PRGs happens, it means many layers of PRGs will stack together and form multi-layers PRGs. As a result, the adhesion friction forces between pristine graphene sheets and cementitious gels are diminished due to the effects of weak van der Waals bonds among multi-layers PRGs that causes the debonding and displacement between those PRGs during sustaining external loads, resulting in a decrease in their strengths. Based on the SEM images and the above analyses, the crack paths of PRG-cement based composites under external loads are outlined in Fig. 10. The outcomes of these findings have provided the better knowledge of the reinforcing mechanism of PRGs on the strengths of cementitious composites prepared with PRG additive through enhancing the PRG–cementitious gels, load-transfer mechanism, and crack paths of the composites.

## 4. Conclusions

This study has presented the proposed reinforcing mechanism and optimized dosage of pristine graphene additives for improving the mechanical strengths of cementitious mortar composites. The main findings of this study can be drawn below:

The strengths of the mortars are dependent on the PRG dosage and size. The PRG sizes (as changed from 23 μm (S2) to 56 μm (S1)) have a significant effect on the enhancement rates of mechanical strengths of the mortars, whereas they do not have a significant influence on the optimized PRG dosage for mechanical strengths of the mortars. The PRG dosage of 0.07% is identified as the optimized concentration of PRG for enhancing the strength of cementitious mortar composites.

At the optimized dosage of 0.07%, the enhancement rates of 7 day & 28 day compressive strengths and tensile strengths of the mix containing larger PRG size S1 are approximately 3.5 and 2.4 times & 1.2 and 2.1 times more than those of the mix with smaller PRG size S2, respectively. The mortars show less improvement in strengths when PRG is used over the optimal dosages. This is due to van der Waals forces between PRGs, resulting in the agglomeration of PRGs and the formation of multi-layers PRGs, resulting in the hindrance to the enhancement of PRGs to the hydration process.

The reinforcing mechanism of PRG on the strengths of cementitious composites is mostly attributed to the adhesion friction forces between pristine graphene sheets and cementitious gels. This can enhance cementitious gels around PRGs and create interlocked PRG–cementitious gels in a space network structure, resulting in the effectiveness of stress distribution. As a result, the mixes containing the larger PRG size (S1) have higher strength improvements than those containing the smaller PRG size (S2). This is because the larger PRG size has larger interaction areas with CSH gels, leading to larger strengthened areas that will be more beneficial for their interlock in the cement matrix compared to the smaller size.

The results from microstructural analyses have indicated that there is a correlation between the strength of cementitious composites and their microstructures. This shows that the mixes with higher strengths often have better microstructure patterns.

The results of this study have not only provided a better understanding of incorporating PRG into cementitious composites, but they have also shown the great potential for low-cost industrially produced PRG materials for improving the performance of cement-based construction materials. The study has also provided a valuable orientation in studying PRG-cement based composites so that further investigations on other properties of pristine graphene and cementitious materials can be performed with less time and effort and fewer costs.

## Conflicts of interest

There are no conflicts to declare.

## Supplementary Material

RA-010-D0RA07639B-s001
