# Supplementary material for: Investigating the reinforcing mechanism and optimized dosage of pristine graphene for enhancing mechanical strengths of cementitious composites
Source: RSC Adv. 2020 Nov 25;10(70):42777–89. doi: 10.1039/d0ra07639b (PMC9057986; doi:10.1039/d0ra07639b)
Supplement: RA-010-D0RA07639B-s001 [file RA-010-D0RA07639B-s001.pdf]

Electronic Supplementary Material (ESI) for RSC Advances.
This journal is © The Royal Society of Chemistry 2020

Investigating the reinforcing mechanism and optimized dosage of pristine graphene for enhancing mechanical strengths of cementitious composites

Van Dac Ho[a,b,c], Ching-Tai Ng*[a], Togay Ozbakkaloglu[d], Ramesh U. Karunagaran[b,c], Farzaneh Farivar[b,c], Andy Goodwin[e], Craig Mc Guckin[e], Van Duong Ho[f], Dusan Losic*[b,c]

Supporting information

This section includes the schematic mechanism of an electrochemical exfoliation process and typical TGA-DTG curves of graphene oxide (GO) and reduced graphene oxide (rGO).

1. The schematic illustration of an electrochemical exfoliation process of PRG materials

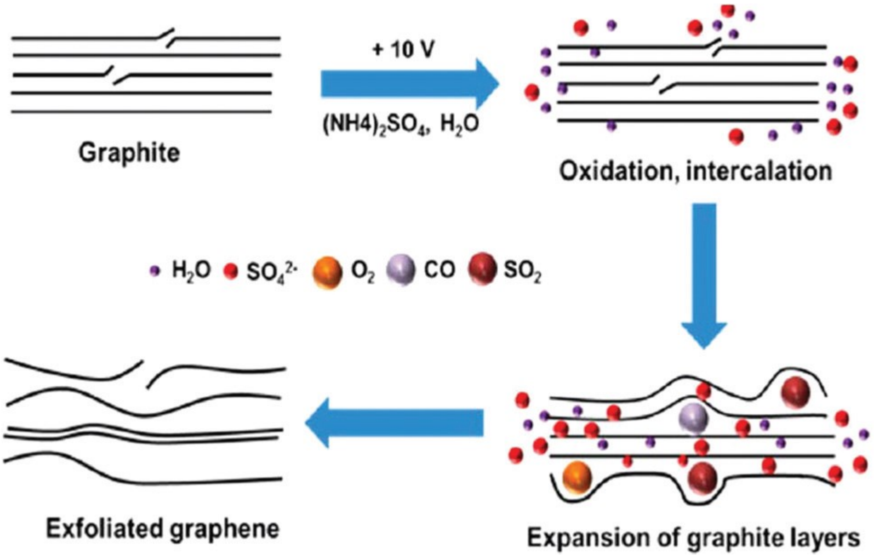

Fig. S1. The general schematic mechanism of PRG materials produced by an electrochemical exfoliation process [1, 2].

[a] School of Civil, Environmental and Mining Engineering, The University of Adelaide, Australia
[b] School of Chemical Engineering, The University of Adelaide, Australia
[c] ARC Research Hub for Graphene Enabled Industry Transformation, The University of Adelaide, Australia
[d] Ingram School of Engineering, Texas State University, United States
[e] First Graphene Ltd, Suite 3, 9 Hampden Road, Nedlands WA 6009, Australia
[f] University of Architecture Ho Chi Minh City
*Corresponding authors: dusan.losic@adelaide.edu.au; alex.ng@adelaide.edu.au

2. Thermogravimetric analysis diagrams of GO and rGO

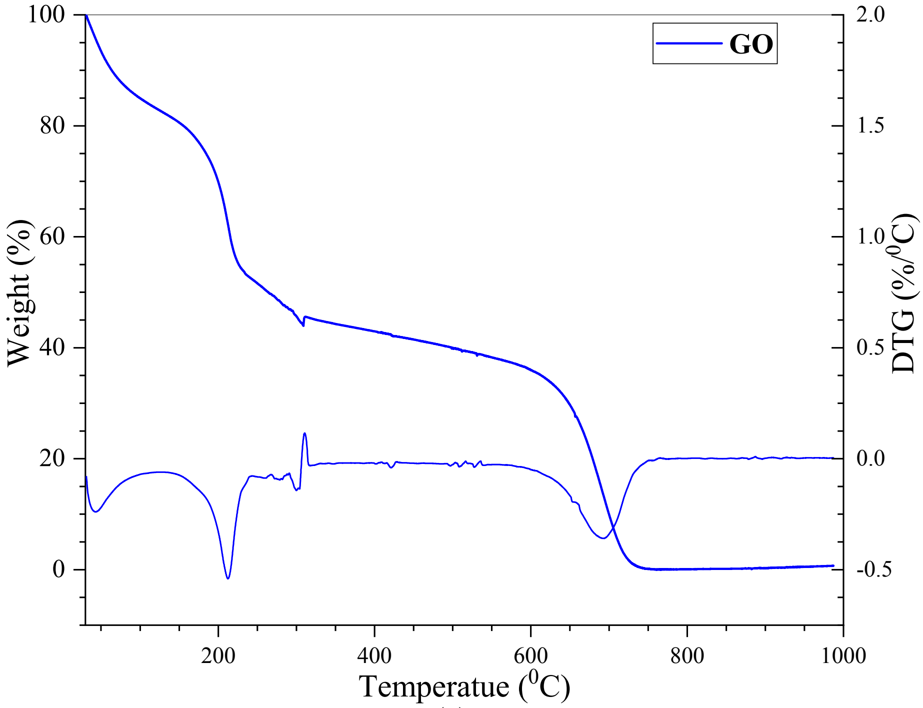

(a)

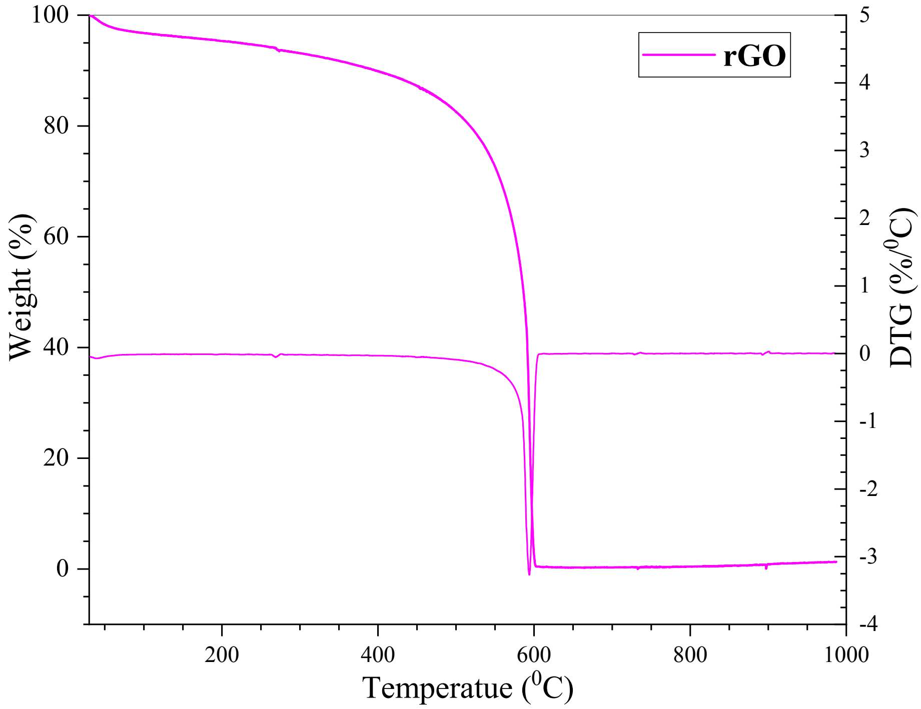

(b)

Fig. S2. Thermogravimetric curves and derivative thermogravimetric curves of GO (a) and rGO (b).

References

1. K. Parvez, Z.-S. Wu, R. Li, X. Liu, R. Graf, X. Feng and K. Mullen, *Journal of the American Chemical Society*, 2014, **136**, 6083-6091.
2. S. Yang, M. R. Lohe, K. Müllen and X. Feng, *Advanced materials*, 2016, **28**, 6213-6221.
